# Graft-derived horizontal cells contribute to host-graft synapses in degenerated retinas after retinal organoid transplantation

**DOI:** 10.1016/j.stemcr.2025.102545

**Published:** 2025-06-26

**Authors:** Mikiya Watanabe, Takayuki Yamada, Hung-Ya Tu, Taro Chaya, Satoko Okayama, Kenta Onoue, Shigenobu Yonemura, Chieko Koike, Masao Tachibana, Takahisa Furukawa, Michiko Mandai

**Affiliations:** 1 Graduate School of Pharmacy, Ritsumeikan University, Kusatsu, Siga 525-8577, Japan; 2 Cell and Gene Therapy in Ophthalmology Laboratory, BZP, RIKEN, Wako, Saitama 351-0198, Japan; 3 Research Center, Kobe City Eye Hospital, Kobe, Hyogo 650-0047, Japan; 4 Laboratory for Retinal Regeneration, RIKEN Center for Biosystems Dynamics Research, Kobe, Hyogo 650-0047, Japan; 5 Laboratory for Molecular and Developmental Biology, Institute for Protein Research, Osaka University, Osaka, Japan; 6 Laboratory for Ultrastructural Research, RIKEN Center for Biosystems Dynamics Research, Kobe, Hyogo 650-0047, Japan; 7 Department of Cell Biology, Tokushima University Graduate School of Medicine, Tokushima 770-8503, Japan; 8 Center for Systems Vision Science, Organization of Science and Technology, Ritsumeikan University, Kusatsu, Shiga 525-8577, Japan; 9 Ritsumeikan Global Innovation Research Organization (R-GIRO), Ritsumeikan University, Kusatsu, Siga 525-8577, Japan; 10 College of Pharmaceutical Science, Ritsumeikan University, Kusatsu, Siga 525-8577, Japan; 11 Laboratory for Animal Resources and Genetic Engineering, RIKEN Center for Biosystems Dynamics Research, Kobe, Hyogo 650-0047, Japan

**Keywords:** retinal organoid, transplantation, multielectrode arrays, ES cells, retinal degeneration, visual function, triad ribbon synapse, horizontal cells

## Abstract

Stem cell-derived retinal organoid (RO) transplantation is a promising approach for treating retinal degenerative diseases such as retinitis pigmentosa. Photoreceptors (PRs) in RO sheets form synaptic connections with host bipolar cells (BCs) and improve visual function in animal models of advanced retinal degeneration. However, the contribution of horizontal cells (HCs), which are critical for PR synapse formation in the developing retina, to host-graft synapse formation remains unclear. In this study, we used HC-depleted retinal degeneration mice (*rd1*) and *Islet1*^−/−^ genome-edited ROs (gROs) that contain HCs but not rod BCs and showed that host HC deficiency did not alter the number of host BC-graft PR synapses after transplantation into *rd1*, while the restored light sensitivity was enhanced in the context of host HCs. These findings indicate that graft-derived HCs alone can support host-graft synapse formation, whereas the presence of host HCs facilitates functional recovery after gRO transplantation.

## Highlights

• Horizontal cells (HCs) degenerate unevenly after photoreceptor loss in *rd1* mice
• Photoreceptors in bipolar-KO retinal organoids utilize graft HCs for integration
• Bipolar-KO retinal organoids (gROs) elicit light responses in HC-KO host *rd1* retinas
• Photoreceptors and HCs in gROs form triad synapses with bipolar cells in HC-KO *rd1*

## Graphical abstract

**Figure:**
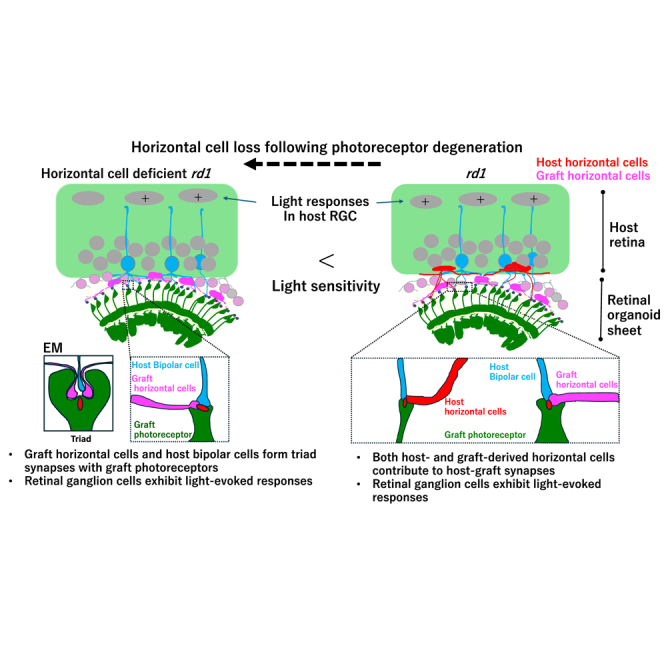

## Teaser

Mandai and colleagues assessed the role of horizontal cells (HCs) in photoreceptor integration following bipolar cell-deficient retinal organoid transplantation in advanced retinal degeneration. They demonstrate that graft HCs can drive host-graft synapse formation, while residual host HCs significantly amplify visual recovery. These findings provide significant insights for optimizing the clinical translation of stem cell therapies for retinal degeneration.

## Introduction

Stem cell-derived photoreceptor (PR) transplantation represents an emerging therapeutic approach for the treatment of retinal degenerative diseases, such as retinitis pigmentosa. Recent advances demonstrate that retinal organoid (RO) sheets, derived from either embryonic stem cells or induced pluripotent stem cells (iPSCs), can establish functional synaptic connections with host bipolar cells (BCs) following transplantation. These connections elicit light responses in *rd1* mice, which serve as a well-established model of advanced retinal degeneration (Mandai et al., 2017; Matsuyama et al., 2021; Akiba et al., 2024a). Building upon promising follow-up studies using human iPSC-derived ROs (Watari et al., 2023), researchers at Kobe City Eye Hospital conducted a clinical investigation evaluating the safety profile of human iPSC-derived RO sheets in patients with retinitis pigmentosa (Hirami et al., 2023). Their findings confirmed the safety and stable survival of transplanted sheets over 2 years in two patients with advanced retinal degeneration. However, additional research is essential to elucidate the precise mechanism underlying the functional integration of graft-derived PRs and to enhance the overall efficacy of RO transplantation.

In the developing mammalian retina, BCs and horizontal cells (HCs) invaginate PR terminals toward synaptic ribbons, creating a distinctive triad synaptic structure (Haverkamp et al., 2000). This developmental process relies heavily on HCs, which guide the dendrites of rod BCs (RBCs) and ON-cone BCs to their respective rod and cone terminals (Nemitz et al., 2019; 2021). Recent electron microscopy studies using correlative array tomography have revealed that host BC dendrites invaginate the synaptic ribbons of graft PRs along with HCs (Akiba et al., 2024a). However, the presence of both graft and host HCs in RO-transplanted retinas leaves ambiguity regarding which HC population primarily contributes to this critical step in host-graft synapse formation.

Following PR degeneration, retinal cells in the inner nuclear layer (INL), including HCs, undergo secondary degeneration (Strettoi and Pignatelli, 2000; Kalloniatis et al., 2016), potentially impacting host-graft synapse formation after RO transplantation. Our previous research using genome-edited ROs (*Islet1*^−/−^, gROs) lacking RBCs demonstrated an increase in host-graft synapses per host L7-GFP-positive RBCs, although the ratio of L7-GFP-positive RBCs forming new synapses with graft-derived PRs to total L7-GFP-positive RBCs remained constant (Matsuyama et al., 2021). gRO-transplanted retinas exhibited enhanced light sensitivity compared to non-gROs (Matsuyama et al., 2021; Yamasaki et al., 2022) and achieved sophisticated light-adapted visual processing with millisecond time resolution even in severely degenerated *rd1* retinas (Watanabe et al., 2025). Despite these advancements, post-transplantation synaptic reconstruction displays considerable heterogeneity, as evidenced by the variable regrowth patterns of L7-GFP-positive RBC dendrites forming *de novo* synapses among PR rosettes in transplanted *rd1* (TP-*rd1*) retinas (Akiba et al., 2024a). These observations strongly suggest that host-side environmental factors significantly influence the regional variability in RO integration, leading us to hypothesize that differences in host-graft synapse formation may be attributed to varying degrees of HC degeneration across retinal regions.

This study investigates the secondary changes in HCs within *rd1* retinas and examines their functional role in host-graft synapse reconstruction. We first characterized the age-dependent decrease in HC population in *rd1* mice. Subsequently, using the RBC reporter in host mice (*rd1*/L7-GFP/*Cx57*-tdTomato) and the synapse reporter (*Nrl*-CtBP2:tdTomato) in gRO grafts (Matsuyama et al., 2021), we assessed whether PR ribbons in the gRO at host-graft synaptic sites could establish connections with either host or graft HCs. To create an extreme-end model, we transplanted gROs into HC-deficient retinal degeneration (*rd1*-dHC) mice using the *Cx57*-Cre system, which specifically induces diphtheria toxin A (DTA) expression in host HCs (Chaya et al., 2017). Through histochemical analyses of transplanted *rd1*-dHC (TP-*rd1*-dHC) mice, we examined synaptic contacts between PRs in the graft and host L7-GFP-positive RBCs. Additionally, we assessed light-evoked responses in host retinal ganglion cells (RGCs) using a multielectrode recording system. Our findings revealed that light responses were successfully recovered in TP-*rd1*-dHC retinas, although with reduced sensitivity compared to TP-*rd1* retinas. Electron microscopy further demonstrated that graft HCs and host BCs contributed to the formation of triad synapses with graft-derived PRs, even in the absence of host HCs. These results collectively indicate that host-graft synapses can be established with gRO-derived HCs alone, although gRO transplantation exhibited better function in the presence of surviving host HCs in *rd1* retinas.

## Results

### Progressive degeneration of HCs following PR loss in retinas of *rd1* mice

*Cx57* is exclusively expressed in HCs of the retina (Hombach et al., 2004). We used *Cx57*-Cre mice (Chaya et al., 2017) for genetic manipulation of HCs. To evaluate the survival rate of HCs in PR-degenerated retinas, we generated *Cx57*-tdTomato mice (*Cx57*-Cre mice × Ai9 mice) and *rd1*/*Cx57*-tdTomato (*Cx57*-tdTomato mice × *rd1* mice) to selectively express tdTomato (Table S1). We confirmed that tdTomato was co-expressed in calbindin-positive HCs along the outer plexiform layer (Figures S1A–S1D). Calbindin-positive but tdTomato-negative cells were observed in the INL and ganglion cell layer, likely representing subsets of amacrine cells and RGCs (Haverkamp and Wässle, 2000; Gu et al., 2016).

**Figure 1:**
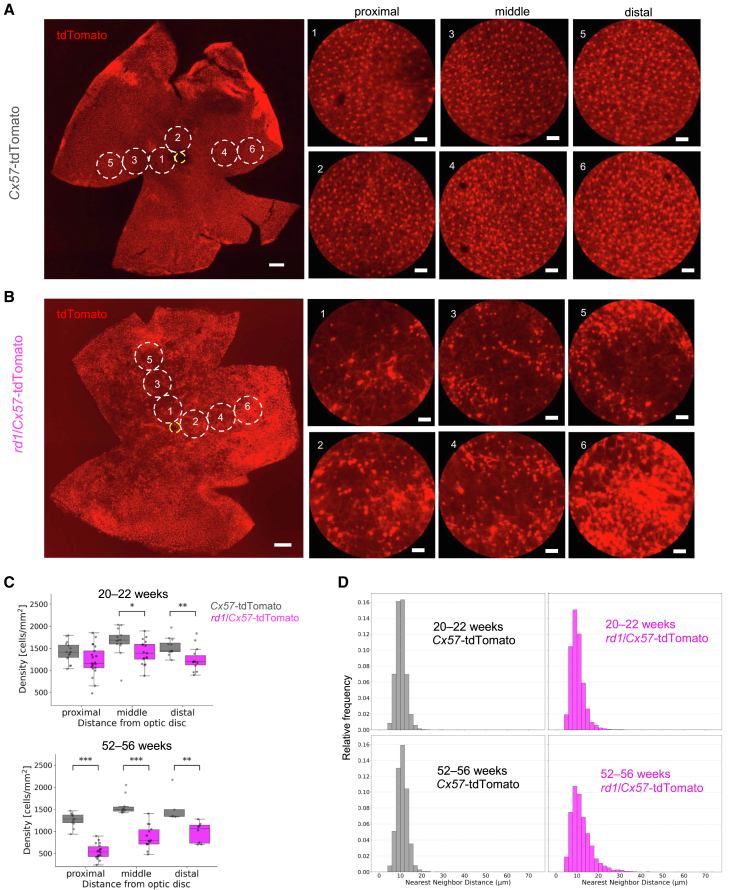
Assessment of horizontal cell degeneration in *Cx57*-tdTomato and *rd1*/*Cx57*-tdTomato retinas (A and B) Representative retinas from *Cx57*-tdTomato (52 weeks; A) and *rd1*/*Cx57*-tdTomato (53 weeks; B) mice. *Cx57*-tdTomato was specifically expressed in horizontal cells (HCs). On the right, the regions marked by circles 1–6 are magnified and categorized as proximal, middle, or distal relative to the optic disc. The yellow circle indicates the optic disc. Scale bars (left image) 300 μm, (right magnified images) 30 μm. (C) Density of HCs at three regions in *Cx57*-tdTomato and *rd1*/*Cx57*-tdTomato retinas at 20- to 22-week-old retinas (top: *Cx57*-tdTomato; 5 retinas from 3 mice, *rd1*/*Cx57*-tdTomato; 6 retinas from 3 mice, middle; *p* = 0.013, distal; *p* = 0.0050) and 52- to 56-week-old retinas (bottom: *Cx57*-tdTomato; 5 retinas from 3 mice, *rd1*/*Cx57*-tdTomato; 5 retinas from 3 mice, proximal; *p* = 5.32 × 10^−6^, middle; *p* = 4.69 × 10^−5^, distal; *p* = 0.0043). Error bars denote standard deviation. (D) Nearest neighbor distance (NND) between HCs was calculated, and its distribution was summarized as a density histogram for each group of retinas (20- to 22-week-old retinas: *Cx57*-tdTomato; 5 retinas from 3 mice, *rd1*/*Cx57*-tdTomato; 6 retinas from 3 mice, 52- to 56-week-old retinas: *Cx57*-tdTomato; 5 retinas from 3 mice, *rd1*/*Cx57*-tdTomato; 5 retinas from 3 mice). The *y* axis represents the relative frequency of NNDs in each group.

In *rd1* retinas, rod PRs undergo nearly complete degeneration by approximately 4 weeks of age (Fujii et al., 2016; Matsuyama et al., 2021). We examined HC density across various retinal regions (Figures 1A, 1B, and S2). In 20- to 22-week-old *rd1*/*Cx57*-tdTomato retinas, HC density decreased in a heterogeneous pattern (Figures 1C and S2). In 52- to 56-week-old *rd1* retinas, HC density significantly decreased across all analyzed regions, with patchy HC defects being more evident than in 20- to 22-week-old *rd1* retinas (Figures 1A, 1B, and 1C). To quantify this spatial heterogeneity in HC degeneration, we measured their nearest neighbor distance (NND) and generated a density histogram of NNDs (Figure 1D). High NNDs occurred more frequently in *rd1*/*Cx57*-tdTomato retinas than in *Cx57*-tdTomato retinas, although the minimum NND remained similar between *rd1*/*Cx57*-tdTomato and *Cx57*-tdTomato retinas (Figure 1D). These findings demonstrate that HC degeneration progresses in a spatially uneven manner following PR degeneration in the *rd1* retina.

### Potential contribution of host- and graft-derived HCs in *rd1* retinas following gRO sheet transplantation

**Figure 2:**
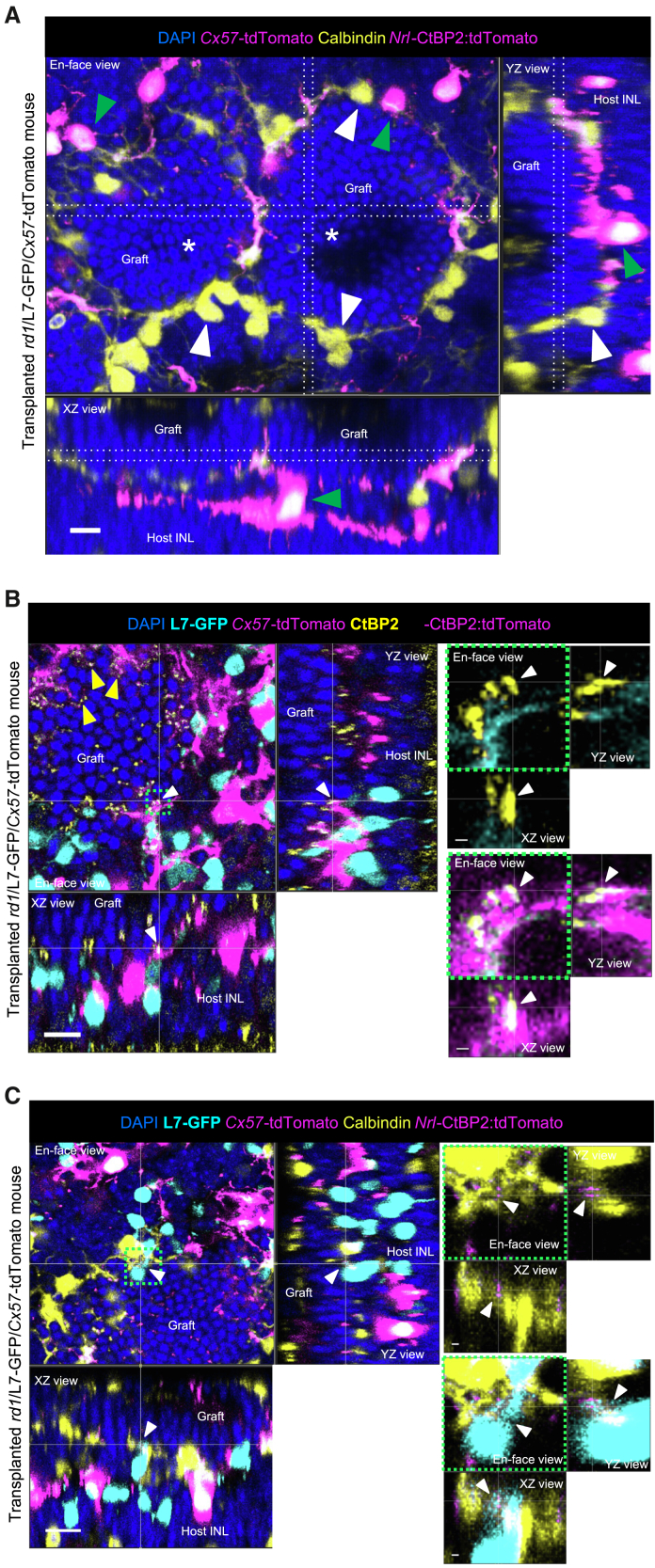
Contribution of host and graft horizontal cells to host-graft synapse reconstruction in *rd1*/L7-GFP/*Cx57*-tdTomato retinas following gRO transplantation (A) Calbindin-positive (yellow), tdTomato-negative graft horizontal cells (HCs, white arrowheads) were observed surrounding the graft photoreceptor rosettes (asterisks). Host HCs co-labeled with *Cx57*-tdTomato fluorescence and calbindin immunoreactivity (green arrowheads) mingled among graft HCs. The white dotted lines in each dimensional view (XY, XZ, YZ) indicate the corresponding positions where the maximum intensity projections were generated along the perpendicular cross-sectional planes. Scale bars: 10 μm. (B) *Nrl*-CtBP2:tdTomato-positive synaptic ribbons (co-labeled with yellow) were in contact with host HCs (magenta) and L7-GFP-positive rod bipolar cells (RBCs, cyan, white arrowheads). CtBP2-positive synaptic ribbons (yellow) were often observed in the processes of *Cx57*-tdTomato-positive HCs (magenta) (yellow arrowheads). Note that *Nrl*-CtBP2:tdTomato fluorescence is much weaker than *Cx57-*tdTomato. A square region surrounded by a dotted green line (left en face view) was magnified on the right side. Scale bars (left images) 10 μm, (magnified images) 1 μm. (C) *Nrl*-CtBP2:tdTomato-labeled synaptic ribbons (magenta) are in contact with graft HC processes (yellow) and L7-GFP-positive host RBCs (cyan) where host *Cx57*-tdTomato-positive HCs are absent (white arrowheads). Magnified images are shown on the right side. Scale bars (left images) 10 μm, (magnified images) 1 μm. INL, inner nuclear layer.

Previous studies have shown that gRO-transplanted *rd1* retinas exhibited enhanced host-graft synapse formation compared to non-edited RO-transplanted *rd1* retinas, with both demonstrating local field potentials and RGC firing responses to light stimulation (Matsuyama et al., 2021). To investigate the contribution of residual host HCs and graft-derived HCs to host-graft synapses, RO sheets lacking RBCs by deletion of *Islet1* gene (gROs, Figure S3) were transplanted into *rd1*/L7-GFP/*Cx57*-tdTomato mice with well-advanced retinal degeneration (Table S2). Histological analyses were performed more than 6 weeks after transplantation. Consistent with previous reports (Mandai et al., 2017; Matsuyama et al., 2021; Watanabe et al., 2025), the transplanted gRO sheets formed PR rosette-like structures of varying complexity (Figure 2A, asterisks). Calbindin-positive graft-derived HCs surrounded each PR rosette in gRO, where host HCs distinguished by the co-labeling of calbindin and tdTomato intermittently mingled with these graft-derived HCs (Figure 2A). The synaptic ribbon marker CtBP2 was observed in gRO PRs contacting the tips of L7-GFP-positive host RBC dendrites (Figure 2B), with host HC processes present at some of these synaptic contact sites, suggesting host HC involvement in host-graft synapse reconstruction. Additionally, calbindin-positive/tdTomato-negative HC processes from the graft gRO were also observed at contact sites between *Nrl*-CtBP2:tdTomato synaptic ribbons and L7-GFP-positive host RBC dendrites (Figure 2C). These findings indicate that both host- and graft-derived HCs potentially contribute to the reconstruction of host-graft synapses in transplanted TP-*rd1* retinas.

### Partial restoration of RGC light responses in HC-deficient *rd1* retinas following gRO transplantation

**Figure 3:**
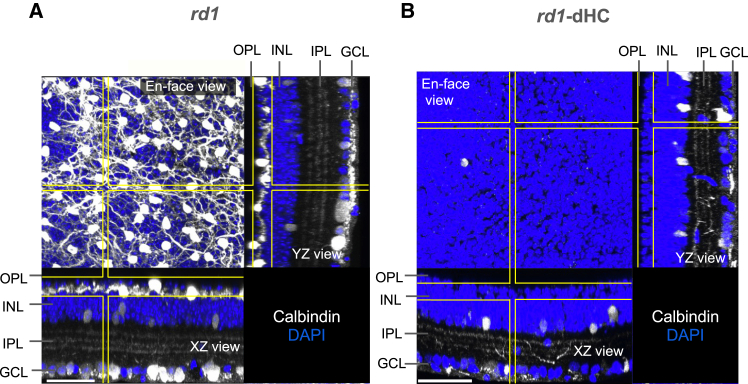
Selective elimination of horizontal cells in *rd1* retinas (A) In the *rd1* retina, calbindin-positive horizontal cells (HCs) were observed. The yellow lines in each dimensional view (XY, XZ, YZ) indicate the corresponding positions where the maximum intensity projections were generated along the perpendicular cross-sectional planes. (B) In the *rd1*-dHC retina, calbindin-positive HCs were absent. Scale bars: 50 μm. OPL, outer plexiform layer; INL, inner nuclear layer; IPL, inner plexiform layer; GCL, ganglion cell layer.

To further elucidate the specific contribution of graft-derived HCs, we developed an end model by generating transgenic mice expressing CreERT2 and DTA under the *Cx57* promoter to selectively eliminate HCs in the host retinas (Chaya et al., 2017). These mice received a tamoxifen-supplemented diet for 5 weeks, starting approximately 1 month after birth, to selectively induce DTA expression in the host HCs. Immunostaining of calbindin in whole-mount retinas isolated from these mice in HCs (Table S1) confirmed that calbindin-positive HCs were lined up in the outer part of the INL in the *rd1* retina (Figure 3A) but were absent in the HC-deficient *rd1* (*rd1*-dHC) retina (Figure 3B).

**Figure 4:**
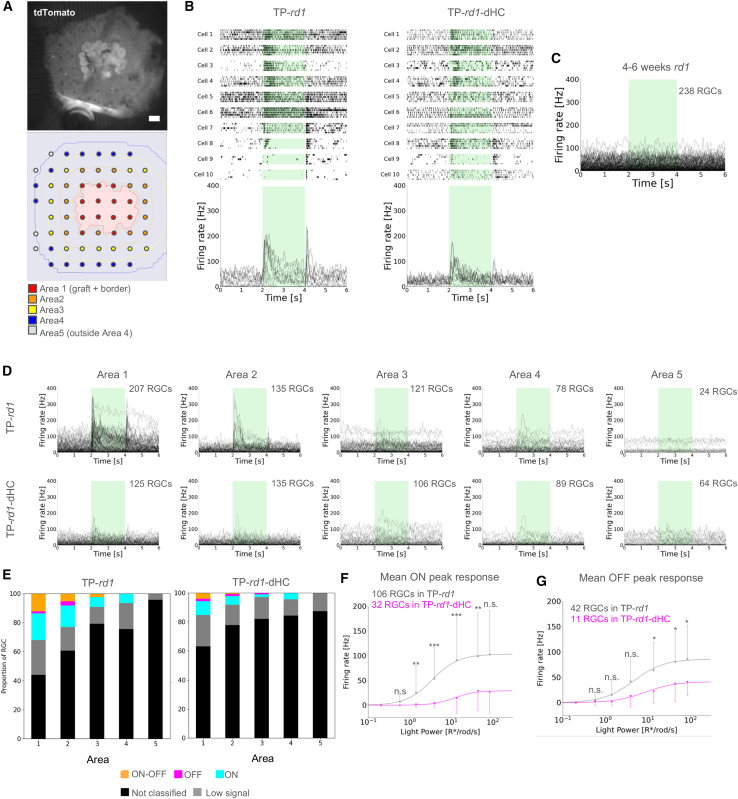
Functional assessment of retinal ganglion cell responses in host *rd1* retinas with or without horizontal cells following gRO transplantation (A) Top view: the TP-*rd1*-dHC retina was placed with the RGC side attached to the MEA, and the grafted area was identified by the presence of *Nrl*-CtBP2:tdTomato fluorescence. Bottom view: MEA areas were divided into area 1 (graft and border) and areas 2–5 as described in methods. Scale bars: 200 μm. (B) Raster plots and PSTHs calculated from 10 representative RGCs from TP-*rd1* (left) and TP-*rd1*-dHC (right) retinas. Green bars from 2 to 4 s indicate 95.23 R^∗^/rod/s 2-s full-field flash stimulation. (C) Superimposed PSTHs of all RGCs in 4- to 6-week-old *rd1* mice (3 retinas, 3 animals). The green bar from 2 to 4 s indicates 95.23 R^∗^/rod/s 2-s full-field flash stimulation. (D) Superimposed PSTHs of all RGCs from areas 1 to 5 in TP-*rd1* (9 retinas, 9 animals) and TP-rd1-dHC (7 retinas, 7 animals) retinas. Green bars from 2 to 4 s indicate 95.23 R^∗^/rod/s 2-s full-field flash stimulation. (E) Classification of RGC response types (same in D) into “ON,” “OFF,” “ON-OFF,” “Low signal,” and “Not classified” in TP-*rd1* (9 retinas, 9 animals, 565 RGCs) and TP-*rd1*-dHC (7 retinas, 7 animals, 519 RGCs) retinas. The number of each RGC type is listed in Table S3. (F and G) Relationship between the mean firing rate of peak responses and light intensity was analyzed in TP-*rd1* (9 retinas, 9 animals) and TP-*rd1*-dHC (7 retinas, 7 animals) retinas. ON responses (F: TP-*rd1*; 106 RGCs, TP-*rd1*-dHC; 32 RGCs, 1.49 R^∗^/rod/s; *p* = 0.0094, 4.32 R^∗^/rod/s; *p* = 3.08 × 10^−5^, 15.32 R^∗^/rod/s; *p* = 5.51 × 10^−7^, 49.38 R^∗^/rod/s; *p* = 0.0060) and OFF responses (G: TP-*rd1*; 42 RGCs, TP-*rd1*-dHC; 11 RGCs, 15.32 R^∗^/rod/s; *p* = 0.030, 49.38 R^∗^/rod/s; *p* = 0.030, 95.23 R^∗^/rod/s; *p* = 0.045). Error bars denote standard deviation.

Six weeks or later after gRO transplantation into 10- to 20-week-old *rd1* and *rd1*-dHC mice (Table S2), we applied a multielectrode array (MEA) system to the isolated retinas to record spike discharges from RGCs. The grafts showed stable survival in both host retina types, with the grafted region identified by *Nrl*-CtBP2:tdTomato fluorescence expressed at the PR terminals (Figures 4A top and S4). We mapped the margins of the PR rosettes in the graft onto the MEA electrodes as area 1 (Figure 4A, bottom, red) and further subdivided the surrounding region into areas 2–5 (Figure 4A, bottom). Flash stimulation was applied under dark background conditions, and we generated raster plots and peri-stimulus time histograms (PSTHs) after spike sorting (Figures 4B and 4C). Light-evoked responses were successfully recorded from some RGCs in both TP-*rd1* (*n* = 9) and TP-*rd1*-dHC (*n* = 7) retinas (Figure 4B; Table S2), whereas no light-evoked responses were detected in *rd1* retinas (*n* = 3) at 4–6 weeks of age within the same light intensity range (Figure 4C; Table S1).

The light-evoked responses recorded from areas 1–5 were superimposed (Figure 4D) and then were classified into “ON,” “OFF,” “ON-OFF,” “Low signal,” and “Not classified” response types (Figure 4E; Table S3). The ratio of light-responsive to non-responsive RGCs was lower in TP-*rd1*-dHC retinas than in TP-*rd1* retinas. We analyzed the relationship between the peak firing rate and light intensity (Figures 4F and 4G): peak firing rates were measured from ON responses of the ON and ON-OFF RGCs (Figure 4F) and OFF responses of the OFF and ON-OFF RGCs (Figure 4G). Both ON and OFF responses in TP-*rd1*-dHC retinas exhibited significantly reduced peak firing rates compared to TP-*rd1* retinas. Furthermore, the peak firing rate-light intensity curve shifted toward higher light intensities in TP-*rd1*-dHC retinas than in TP-*rd1* retinas, suggesting that light sensitivity was lower in the former than in the latter. These findings demonstrate that while retinal function can be restored in TP-*rd1*-dHC retinas lacking host HCs, the presence of host HCs appears to enhance retinal light sensitivity following gRO transplantation.

### Graft-derived HCs contribute to synaptic reconstruction in the absence of host HCs

**Figure 5:**
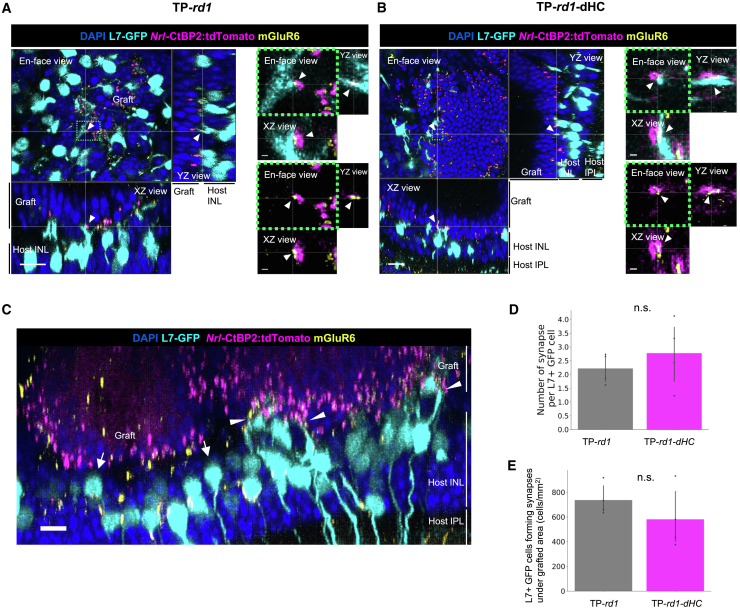
Quantitative analyses of host-graft synaptic contacts in host *rd1* retinas with or without horizontal cells following gRO transplantation (A and B) L7-GFP-positive host rod bipolar cells (RBCs, cyan) were colocalized with graft photoreceptor (PR) synaptic ribbons (*Nrl*-CtBP2:tdTomato, magenta) and postsynaptic marker mGluR6 (yellow) in TP-*rd1* (A) and TP-*rd1*-dHC (B) retinas. Scale bars (left) 15 μm, (magnified images on the right side) 1 μm. (C) L7-GFP-positive host RBCs in the TP-*rd1* retina exhibit variability in dendritic regrowth, forming *de novo* synapses actively in some PR rosettes (white arrowhead: abundant contacts) while showing limited or no regrowth in others (white arrow: sparse contacts). Scale bars: 10 μm. (D) Number of synapses per L7-GFP-positive host RBCs was quantified within the graft PR rosettes of TP-*rd1* (4 retinas) and TP-*rd1*-dHC retinas (4 retinas). Synapse was defined as a CtBP2 spot within 1.5 μm from an mGluR6 spot and an RBC dendrite. (E) Density of L7-GFP-positive RBCs forming synapses under the graft, calculated as the number per unit grafted area in TP-*rd1* (4 retinas) and TP-*rd1*-dHC retinas (4 retinas). Error bars denote standard deviation. INL, inner nuclear layer.

We examined host-graft synapse formation at least 6 weeks after transplantation using the RBC reporter (L7-GFP) in host retinas and the synaptic reporter (*Nrl*-CtBP2:tdTomato) in gROs (Table S2), combined with immunostaining of the postsynaptic marker mGluR6. In TP-*rd1*-dHC retinas, similar to TP-*rd1* retinas, we identified mGluR6 puncta at the dendritic tips of L7-GFP-positive host RBCs in contact with *Nrl*-CtBP2:tdTomato-positive synaptic ribbons at the PR terminals within the graft, defining these as host-graft synaptic contacts (Figures 5A and 5B). Within individual host retinas, L7-GFP-positive RBCs exhibited variable dendritic regrowth, resulting in heterogeneous synaptic connectivity among the PR rosettes. Certain PR rosettes formed abundant synaptic contacts with RBCs, whereas the others established only sparse contacts (Figure 5C). Quantitative analysis of host-graft synaptic contacts per L7-GFP-positive RBC revealed no significant difference between TP-*rd1* and TP-*rd1*-dHC retinas (Figures 5D and S5). The density of L7-GFP-positive RBCs forming synapses within the grafted PR area did not differ significantly between TP-*rd1* and TP-*rd1*-dHC retinas, suggesting that the absence of host HCs did not affect host-graft synapse formation (Figure 5E).

**Figure 6:**
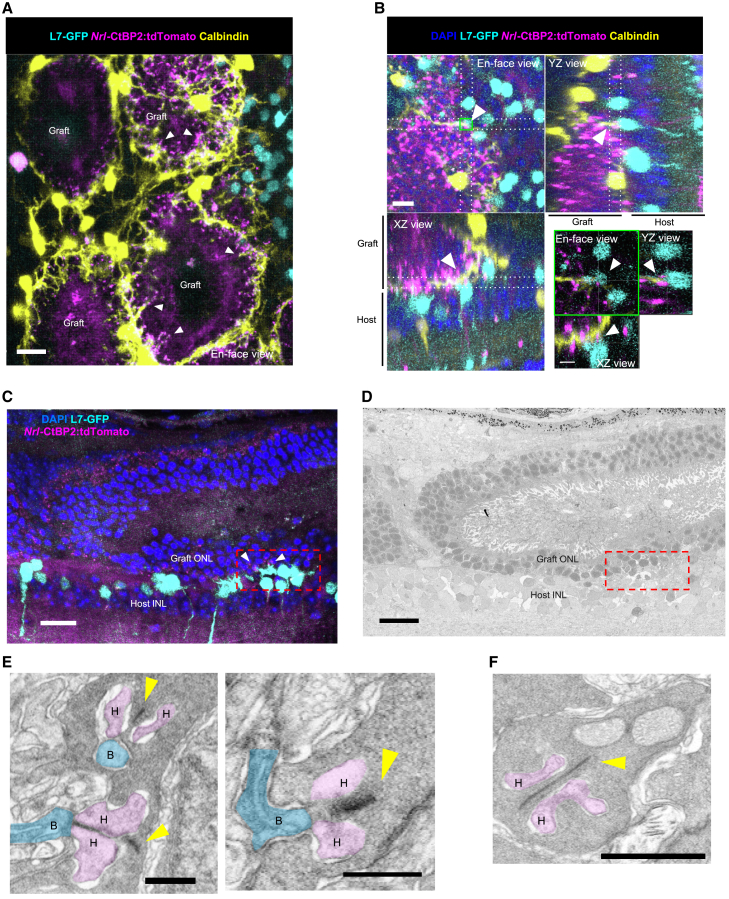
Contribution of graft horizontal cells to host-graft synapse formation in horizontal cell-deficient host *rd1* retinas following gRO transplantation (A) In the TP-*rd1*-dHC retina, graft HCs (yellow) were observed consistently surrounding the photoreceptor (PR) rosette structures. PR synaptic ribbons (*Nrl*-CtBP2:tdTomato, magenta) were frequently colocalized with HC processes (white arrowheads). Scale bars: 15 μm. (B) In TP-*rd1*-dHC retina, L7-GFP-positive host rod bipolar cells (RBCs) contacted graft HCs and PR synaptic ribbons (*Nrl*-CtBP2:tdTomato), indicating the contribution of graft HCs to host-graft synapse formation (white arrowheads). Scale bars: 10 μm, (magnified images) 5 μm. (C) Fluorescent image of a 50-μm-thick TP-*rd1*-dHC retinal section. Host-graft synapses were identified as contact sites between L7-GFP host RBC dendrites and *Nrl*-CtBP2:tdTomato-labeled graft PR ribbons (white arrowheads). Scale bars: 20 μm. (D) Electron microscopic image of serial sections from (C). Scale bars: 20 μm. (E) In the neighboring sections of the boxed area in (D), graft HCs (H, pink) and host BCs (B, blue) form triads at the ribbons (yellow arrows) in graft PRs. Scale bars: 500 nm. (F) Predominant synapses are dyads outside the reach of the host BCs, consisting of synaptic ribbons (yellow arrowheads) and HC processes (H, pink). Scale bars: 1 μm. INL, inner nuclear layer; H, horizontal cell; B, bipolar cell.

We further investigated whether calbindin-positive graft-derived HCs contribute to the formation of host-graft synapses in TP-*rd1*-dHC retinas. Calbindin-positive HC processes within the graft were frequently associated with *Nrl*-CtBP2:tdTomato synaptic ribbons in the area away from the host-graft interface, suggesting the preceding invagination of graft HCs toward graft PR ribbons when RBCs were missing (Figure 6A). At the host-graft synaptic site, where *Nrl*-CtBP2:tdTomato-positive synaptic ribbons were localized at the dendritic tips of L7-GFP-positive host RBCs, graft-derived calbindin-positive HCs were also observed (Figure 6B). To examine the ultrastructure of host-graft synapses in the TP-*rd1*-dHC retina, we first captured a fluorescent image of a retinal section to localize the putative synaptic contact sites (Figure 6C). We then performed electron microscopy on serial sections from the same sample to confirm the presence of synaptic structures at these prefocused host-graft synapse sites (Figure 6D). Triad synapses involving RBCs and HCs were observed in the area where the presence of host-graft synapses was suggested by the reporter fluorescence (Figure 6E). In other areas, HC invagination was consistently observed at the synaptic ribbons of the graft PRs even in the absence of RBC invagination (Figure 6F). Since gRO grafts lack RBCs, these host-graft triads likely consist of graft-derived HCs and host RBCs, demonstrating that graft-derived HCs alone can support the functional integration of graft PRs into the host retina.

## Discussion

During the developmental process of synapse formation between PRs and BCs, HCs play a crucial pioneering role by first contacting and invaginating PR terminals, which is subsequently followed by the invagination of BC processes to form a typical triad (Sherry et al., 2003). Previous studies have demonstrated that selective removal of HCs nearly abolishes the invagination of BC processes into PR terminals, providing compelling evidence that HC invagination serves as an essential guide for BC invagination during synaptogenesis (Nemitz et al., 2019; 2021). In this study, we transplanted *Islet1*-deficient retinal gRO into both standard retinal degeneration mice (*rd1*) and HC-depleted retinal degeneration mice (*rd1*-dHC) to systematically investigate the respective contribution of host or graft HCs to the reconstruction of host-graft synapses. Our findings first established that in *rd1* retinas, following primary PR degeneration, HC populations gradually decline as part of a progressive secondary inner retinal degeneration process. Through detailed histological analyses of TP-*rd1* and TP-*rd1*-dHC retinas, we identified that both the remaining host HCs and graft-derived HC contribute to host-graft PR synapses. Furthermore, graft-derived HC processes frequently associate with synaptic ribbons of rod PRs within the graft in regions beyond the reach of host BCs in TP-*rd1*-dHC mice. This observation strongly indicates that graft-derived HC invagination of PR axon terminals occurs preemptively within gROs, effectively preparing the synaptic microenvironment for subsequent BC invagination. This interpretation was further supported by electron microscopy evidence revealing dyad structures (consisting of PR ribbon and HC processes without BC processes) within the PRs of gRO grafts, representing an intermediate stage of synaptic assembly. The quantitatively similar degree of host-graft synaptic contact observed in both TP-*rd1* and TP-*rd1*-dHC retinas suggests that graft-derived HCs can effectively facilitate the initial process of host-graft synapse formation, even in the absence of host HCs. To assess functional outcomes, we used MEA recordings, which revealed that host RGCs in TP-*rd1*-dHC retinas successfully regained light responsiveness, albeit with reduced sensitivity compared to those in TP-*rd1* retinas. Collectively, these results suggest that transplantation of gROs has the potential to contribute to functional recovery even in advanced disease stages characterized by significant HC loss after PR degeneration.

Despite the comparable number of host-graft synapses observed in both TP-*rd1* and TP-*rd1*-dHC, RGC light responses were reduced in the latter. Several mechanisms may account for this functional discrepancy. First, synaptic maturity or quality may fundamentally differ in the presence versus absence of host HCs. For instance, host BCs that have undergone acute HC deprivation due to DTA-induced cell death may exhibit compromised dendritic rewiring capabilities. Alternatively, host BC dendrites might be more efficiently guided by host HCs than by graft HCs, resulting in more functionally optimized synapse formation. Another possibility involves alterations in the inner retinal circuitry following HC loss. Extensive research with animal models of PR degeneration has demonstrated that inner retinal remodeling begins when PRs start to degenerate (Kalloniatis et al., 2016) and that PR loss can cause synaptic remodeling in the inner plexiform layer (Akiba et al., 2024b). The additional loss of HCs by DTA in *rd1* retinas may have caused further deterioration of the remaining retinal network in *rd1*-dHC retinas, which may have resulted in reduced retinal sensitivity in TP-*rd1*-dHC retinas compared with TP-*rd1* retinas.

In clinical retinal degeneration conditions such as retinitis pigmentosa, HC reduction likely occurs as a secondary consequence of PR loss, as observed in *rd1* retinas. Our findings indicate that gRO transplantation can contribute to the restoration of visual function, even in advanced cases with progressive HC depletion. However, optimal functional recovery may be achieved when gROs are transplanted while sufficient host HCs remain viable. Advanced retinal imaging technologies that enable detailed visualization of remaining retinal cell populations in patients with retinitis pigmentosa may provide valuable predictive information for estimating the efficacy of stem cell-based therapeutic approaches, including gRO transplantation.

### Study limitations

This study suggests a potential contribution of HCs to host-graft synaptic reconstruction. However, to fully understand the significance of host- or graft-derived HCs, further studies are needed in cases where HCs are absent in either the graft alone or in both the graft and host. Although we observed similar densities of host-graft synaptic contacts in TP-*rd1* and TP-*rd1*-dHC retinas, the quality of synaptic transmission in the newly formed host-graft synapses is not yet assessable in our current setup. Additionally, HC deletion and PR degeneration may affect the remaining host retinal network. Therefore, it is unclear whether the reduced light sensitivity observed in TP-*rd1*-dHC retinas, compared to TP-*rd1* retinas, could result from either impairment of newly formed host-graft synapses or disruption of the host retinal network.

## Methods

### Animals

All experimental protocols were approved by the Animal Care Committee of the RIKEN Center for Biosystems Dynamics Research and were conducted in accordance with local guidelines and the ARVO statement on the use of animals in ophthalmic and visual research. The progressive retinal degeneration mouse line C57BL/6J-Pde6b^*rd1−2J*^/J (JAX stock #004766, referred to as *rd1* mice) was crossed with B6; FVB-Tg(Pcp2-EGFP)2Yuza/J (JAX stock #004690, referred to as L7-GFP mice) (Tomomura et al., 2001) mice to produce *rd1/*L7-GFP mice expressing EGFP in RBCs. To genetically target the HCs in the retina, we used BAC-*Cx57*-CreERT2 mice (referred to as *Cx57*-Cre mice) (Chaya et al., 2017). *Cx57* is expressed only in retinal HCs and the thymus medulla (Hombach et al., 2004; Tykocinski et al., 2010). To fluorescently label HCs in *rd1/*L7-GFP retinas, we generated *rd1*-*Cx57*-tdTomato mice by crossing *rd1/*L7-GFP mice with *Cx57*-Cre mice and B6.Cg-Gt(ROSA)26Sortm9(CAG-tdTomato)Hze/J mice (JAX Stock #007909, referred to as Ai9 mice). To selectively remove HCs from *rd1* retinas, we generated *rd1*-dHC mice by crossing *rd1/*L7-GFP mice with *Cx57*-Cre mice and NSE-DTA mice (Kobayashi et al., 2013). Cre was induced by feeding mice a tamoxifen-containing diet for 5 weeks, starting approximately 1 month after birth. Mouse gRO sheets were transplanted subretinally into 10- to 30-week-old *rd1*/L7-GFP, *rd1*/L7-GFP/*Cx57*-tdTomato, and *rd1*-dHC mice. The mice used in each experiment are summarized in Tables S1 and S2.

### Mouse ES cell line and RO differentiation

The mouse embryonic stem (ES) cell line ROSA26^+/*Nrl*−CtBP2:tdTomato^ is a synaptic ribbon reporter line that expresses the *Nrl*-CtBP2:tdTomato fusion protein under the control of the mouse *Nrl* promoter (Akimoto et al., 2006; Mandai et al., 2017). Retinal sheets were prepared for transplantation from the *Islet1*^−/−^ ROSA26^+/*Nrl*−CtBP2:tdTomato^ mouse ES line (Matsuyama et al., 2021). Mouse ES cells were plated at 5,000 cells/well in 96-well plates (Thermo Fisher Scientific, 174925) to form aggregates in differentiation medium (Glasgow minimum essential medium [Gibco, 11710035], 5% KSR [Gibco, 10828-028], 0.1 mM nonessential amino acids [Gibco, 11140-050], 1 mM pyruvate [Sigma, S-8636], and 0.1 mM 2-mercaptoethanol [Wako, 137-06862]) + 100 μM AGN193109 (Toronto Research Chemicals, A427000). The aggregates were incubated at 37°C with 20% O_2_ and 5% CO_2_. On differentiation day (DD) 1, growth factor-reduced Matrigel (2%, BD Biosciences, 354230) was added to the differentiation medium. On DD7, aggregates were transferred to the retinal maturation medium (DMEM/F12 with GlutaMAX [Gibco, 10565], 1% N2 supplement [Gibco, 17502-048], and 1% penicillin-streptomycin [Gibco, 15140-122]) in a 6-cm dish (Corning, 351007) and incubated at 37°C with 40% O_2_/5% CO_2_. On DD11, all-trans retinoic acid (0.5 μM, Sigma, R2625-100MG) and L-taurine (1 mM, Sigma, T8691) were added to the retinal maturation medium. On DD13, a small piece (approximately 1 × 0.5 mm) containing the characteristic continuous neural epithelial structure was excised from each optic vesicle for transplantation (Assawachananont et al., 2014). Representative bright-field images of ROs at DD13 and immunostaining images with markers for retinal neuronal progenitors and other relevant cell types are shown in Figure S3.

### Transplantation of the gRO sheets

Injection tips were prepared from disposable micropipettes (Drummond, 1-000-0500) using a micropipette puller (Sutter Instrument, P-97/IVF Puller), and the tip (∼500 μm in diameter) was sharpened using a microgrinder (Narishige, EG-400). The injection tip was then fixed in a microelectrode holder (World Precision Instruments, MPH310) on the electrode handle (World Precision Instruments, 2505), which was connected to an extension tube, and the route was filled with Hank’s balanced salt solution (Gibco, 14170112) using a 1-mL syringe. Then, the syringe was replaced by a 10-μL micro-syringe (Hamilton, 1701LT), and the gRO sheets were loaded in the injection tip by aspiration with ∼2 μL 6× Viscoat (Alcon) containing hyaluronate and chondroitin sulfate sodium. Mice were anesthetized with a mixture of medetomidine hydrochloride (0.75 mg/kg body weight; Nippon Zenyaku Kogyo Co., Ltd., Domitor), midazolam (4 mg/kg body weight; Maruishi Pharmaceutical, Dormicum), and butorphanol tartrate (5 mg/kg body weight; Meiji Animal Health, Vetorphale. The eyes were dilated with 0.4% tropicamide (Rohto Nitten). Two small scleral punctures were made at the peripheral retina with a 30G needle, one for graft injection and the other for reducing intraocular pressure, and a gRO sheet was gently injected subretinally into the eyes of *rd1* mice.

### Electrophysiology: MEA recordings

Details of spike sorting are described in the supplemental procedures. MEA recordings and analyses were performed using a modified version of the method described in a previous study (Watanabe et al., 2025). Host retinas with engrafted gRO sheets were used for MEA recordings ≥6 weeks after transplantation. The following procedure was performed under a dim light-emitting diode (LED) light with a peak wavelength of 690 nm. After a day of dark adaptation, the mice were anesthetized with isoflurane (Viatris) and sacrificed by cervical dislocation. After enucleation, the cornea and vitreous area were removed, and only the retina was isolated. The transplants were examined visually. The retina was trimmed around the engrafted area to an appropriate size and placed RGC side down onto the MEA electrodes (Multi Channel Systems, 60pMEA200/30iR-Ti: 60 electrodes, electrode size 30 × 30 μm, inter-electrode distance 200 μm). The whole-mount retina was fixed on the electrodes by suction using a vacuum pump, and the retina was constantly perfused with warmed (34°C), carbonated (95% O_2_ and 5% CO_2_) Ames’ medium (Sigma, A1420) at 3–3.5 mL/min. Recordings were started 40 min after perfusion initiation. The extracellular voltage signals were amplified and digitized at 20 kHz using an MEA amplifier (Multi Channel Systems, USB-ME64-System).

### Immunohistochemistry

After MEA recordings, the retina was removed from the MEA, washed with phosphate-buffered saline, and fixed with 4% paraformaldehyde (Wako Pure Chemical Industries, 30525-89-4) for 15 min at room temperature (RT). The retina was then incubated in blocking buffer (3% Triton X-100 and 1% bovine serum albumin [Sigma, A4503]) for 1 h at RT, followed by incubation with primary antibodies—sheep anti-mGluR6 (1:2,000; Leinonen et al., 2020), mouse anti-CtBP2 (1:1,000; BD Biosciences, #6120044), and rabbit anti-calbindin (1:1,000; Millipore, ABN2192)—for 1 week at 4°C. The retina was washed three times with blocking buffer, followed by incubation with secondary antibodies (donkey anti-rabbit Alexa Fluor 647 [1:1,000; Invitrogen, A31573] and donkey anti-mouse Alexa Fluor 647 [1:1,000; Invitrogen, A31571]) and DAPI (1:1,000; Invitrogen, D1306) for 3 days at 4°C.

After incubation with the secondary antibody, the retinas were washed three times with a blocking buffer and mounted on glass slides using Vectashield (Vector Laboratories, H-1000). z stack Images were acquired using a Leica TCS SP8 confocal microscope and reconstructed in three-dimensional imaging using the Imaris Microscopy Image Analysis Software (Oxford Instruments, http://www.bitplane.com/). An overall view of the engrafted whole-mounted retinas was obtained using a BZ9000 microscope (Keyence).

Details of immunostaining of DD13 ROs are described in the supplemental procedures.

### Light simulation for MEA recordings

The light stimulus was generated using an LED with a single peak at 505 nm (Thorlabs, SOLIS-505C), and the entire retina was illuminated through an objective lens. The light intensity was adjusted using an LED modulator (Thorlabs, DC2200) controlled by a function generator (NF Corporation, WF1973). In Figure 4, a 2-s flash is presented under dark background conditions. The flash intensity was changed from 0.83 to 95.22 R^∗^/rod/s.

### MEA analysis

We divided the MEA area into five groups (areas 1–5) according to its distance from the grafted area, which was identified by the presence of *Nrl*-CtBP2:tdTomato fluorescence expressed at the PR terminals of the graft. Area 1 corresponded to the graft, and areas 2–4 referred to areas that gradually expanded away from the graft. To label the regions within the graft and the surrounding area based on their proximity, binary dilation was applied at magnifications of 1×, 5×, 10×, and 15×. Area 5 was defined as being more peripheral than area 4. This approach allowed us to systematically dilate the labeled regions according to their shapes with different grafts, thereby facilitating spatial analysis of the tissue. PSTHs were calculated with a 20-ms bin width and passed through a binomial filter (*n* = 4) for smoothing. Based on the shape of the response to a 2-s flash (95.23 R^∗^/rod/s), each RGC was classified into “ON,” “OFF,” “ON-OFF,” “Low-signal,”, or “Not classified” response types. The mean firing rate was calculated from the spontaneous activity over 10 s in the dark. The threshold was calculated as the mean firing rate +4SD. The response peak was corrected by subtracting the mean firing rate in the dark. When a response peak (>threshold) was detected within 500 ms of light onset, the RGC was classified as ON. When a response peak (>threshold) was detected 50–300 ms after the light offset, it was classified as OFF. When response peaks appeared at light onset and offset, they were classified as ON-OFF. When the peak firing rate (>threshold) was <10 Hz, the signal was classified as low. The remaining RGCs could not be classified. In Figures 4F and 4G, subthreshold peaks were considered as 0 Hz.

The intensity-response curves were fitted using the following equation (Westö et al., 2022):

$$Fit=Rmin+\left(Rmax-Rmin\right)\frac{I^{n}}{I^{n}+{I_{50}}^{n}\;}$$

where *Rmin* and *Rmax* are the lowest and largest peak firing rates, respectively, among all stimulus intensities. *I*_*50*_ is the intensity of the half-maximum response. *I*_*50*_ and *n* values were determined by minimizing the squared error between the measured data and the predicted values of the function.

### Horizontal cell counting

Using a fluorescence microscope (BZ9000), we captured images of whole-mounted retinas from *rd1*/*Cx57*-tdTomato and *Cx57*-tdTomato mice to count the number of HCs fluorescently labeled with tdTomato. The optic nerve head was excluded from the analysis and was used as a reference point. To mark its location, the optic nerve head was enclosed within a circle with a diameter of 100–200 μm.

To investigate the relationship between the distance from the optic nerve head and the number of HCs, we created concentric circles centered on the optic nerve head with diameters of 500 μm, representing proximal, middle, and distal regions. The proximal circle was positioned tangential to the central circle enclosing the optic nerve head, the middle circle was positioned tangential to the proximal circle, and the distal circle was positioned tangential to the middle circle. These circles were aligned along straight lines extending vertically and horizontally from the optic nerve head. Using Cellpose (Stringer et al., 2021), we detected and counted the cell bodies of HCs within each circle. If a region included damaged areas or Cellpose failed to accurately detect cells, the corresponding circle was excluded from the analysis. To quantify the NND, we calculated the Euclidean distance between each cell and its nearest neighbor. We first extracted the *x* and *y* coordinates of all HCs and then used the cKDTree function from scipy.spatial in Python to efficiently identify the nearest neighbor for each cell (excluding the cell itself).

### Host-graft synapse quantification

Host-graft synapses were quantified using a modified version of the method described in our previous study (Akiba et al., 2024a). A new channel was created in the CtBP2 channel using LabKit (Arzt et al., 2022) to trace the entire PR rosette. The “Surface” function in Imaris (Oxford Instruments, https://imaris.oxinst.com/) was then used to trace the rosette and identify L7-GFP-positive host RBCs extending projections within 10 μm distance from the rosette surface. The cell bodies of these RBCs were traced using the “Spot” function and manually reviewed to exclude any apparent errors. Additionally, CtBP2 and mGluR6 puncta were traced using the “Spot” function. Synapse was defined as a CtBP2 spot located within 1.5 μm from the mGluR6 spot and RBC dendrite, determined using the “Shortest distance” function. Finally, RBC dendrites located within 1.5 μm of a synapse were classified as “RBC with forming synapse.” Host-graft synapse identification was reviewed manually to ensure accuracy. An example of synapse detection is shown in Figure S5.

### Electron microscopy

Mice were euthanized via cervical dislocation following isoflurane inhalation anesthesia. Transplanted eyes were fixed in eye cups in a solution containing 4% formaldehyde (Thermo Fisher Scientific, 28908), 2% glutaraldehyde (Wako, 076-02265), 4% sucrose (Nacalai, 30403-55), and 30 mM HEPES (Nacalai, 02443-34) in distilled water at RT for 2 h. The tissues were embedded in agarose gel and sectioned into 50 μm slices using a linear slicer (LinearSlicer PRO7, Dosaka EM Co., Ltd., Kyoto, Japan) and stained with DAPI for capturing fluorescein images for orientation. The sections were washed five times with 0.1 M cacodylate buffer (pH 7.5, Nacalai, 06516-22), fixed in 1% OsO_4_ (TAAB, G017/1) in the same buffer at 4°C for 1 h, and washed five times with distilled water. The specimens were incubated in 1% uranyl acetate overnight and washed five times with distilled water. Dehydration was performed using a graded ethanol series (20%, 50%, 70%, 90%, 99.5%, and 100%, each for 5 min on ice), followed by 100% acetone for 10 min at RT. The samples were infiltrated with Epon812 covered with an Aclar sheet and polymerized at 60°C for 72 h. Ultrathin sections (80 nm) were prepared using an ultramicrotome (EM UC7, Leica Microsystems) equipped with a diamond knife, stained with uranyl acetate and a lead stain solution (Sigma, 18–0875), and examined under a field-emission scanning electron microscope (MERLIN VP compact, Carl Zeiss) at 5 kV using a backscattered electron detector.

### Statistical analysis

In Figures 1C, 4F, 4G, 5D, and 5E, the Mann-Whitney U test was used. The error bars denote the SD. Asterisks in the figures indicate *p* values: ^∗^*p* < 0.05, ^∗∗^*p* < 0.01, and ^∗∗∗^*p* < 0.001.

## Resource availability

### Lead contact

Further information and requests for resources and reagents were directed to the lead contact, Michiko Mandai (e_lab.mandai@kcho.jp).

### Materials availability

The materials used in this study are available from the corresponding author upon request. The request for the BAC-*Cx57*-CreERT2 mice was addressed to Dr. Takahisa Furukawa (takahisa.furukawa@protein.osaka-u.ac.jp).

### Data and code availability

The datasets supporting the current study have not been deposited in a public repository because of the large data size, but they are available from the corresponding author upon request. Additional information required to reanalyze the data reported in this paper is available from the corresponding author upon request.

## Acknowledgments

We thank Junki Sho, Chihiro Hayakawa, Hironobu Syuto, and Toshika Senba at Vision Care Co. for their support with animal experiments. This study was supported by 10.13039/501100001691JSPS KAKENHI 22K09826 (M.M.), 10.13039/501100001691JSPS KAKENHI 20K18403 (H.-Y.T.), Moonshot R&D (JPMJMS2022) (S.Y.), AMED-CREST (21gm1510006) (T.F.), Moonshot R&D (JPMJMS2024) (T.F.), and JST COI-NEXT (JPMJPF2018) (T.F.). We thank Dr. Jeannie Chen (University of Southern California) for providing the mGluR6 antibody and Dr. Shigeyoshi Itohara (RIKEN BSI) for providing the NSE-DTA mice.

## Author contributions

Conceptualization, M.M. and H.-Y.T.; data curation, M.W., T.Y., H.-Y.T., S.O., K.O., and S.Y.; formal analysis, M.W. and T.Y.; funding acquisition, H.-Y.T., T.F., and M.M.; investigation, M.W., T.Y., H.-Y.T., S.O., K.O., and S.Y.; methodology, M.W., T.Y., H.-Y.T., S.O., K.O., S.Y., M.T., and M.M.; project administration, M.M.; resources, H.-Y.T., T.C., T.F., and M.M.; supervision, C.K., M.T., and M.M.; visualization, M.W.; writing – original draft, M.W.; writing – review and editing, H.-Y.T., M.T., and M.M.

## Declaration of interests

M.W. is an employee of VCCT, Inc. T.Y. is an employee of Vision Care, Inc. M.M. is an inventor of patent applications for genetically modified retinal organoids.

## Supplemental information

Document S1. Figures S1–S5 and Tables S1–S3

Document S2. Article plus supplemental information
