# Supplementary material for: Graft-derived horizontal cells contribute to host-graft synapses in degenerated retinas after retinal organoid transplantation
Source: Stem Cell Reports. 2025 Jun 26;20(7):102545. doi: 10.1016/j.stemcr.2025.102545 (PMC12277844; doi:10.1016/j.stemcr.2025.102545)
Supplement: Document S1. Figures S1–S5 and Tables S1–S3 [file mmc1.pdf]

Stem Cell Reports, Volume *20*

Supplemental Information

Graft-derived horizontal cells contribute to host-graft synapses in degenerated retinas after retinal organoid transplantation

Mikiya Watanabe, Takayuki Yamada, Hung-Ya Tu, Taro Chaya, Satoko Okayama, Kenta Onoue, Shigenobu Yonemura, Chieko Koike, Masao Tachibana, Takahisa Furukawa, and Michiko Mandai

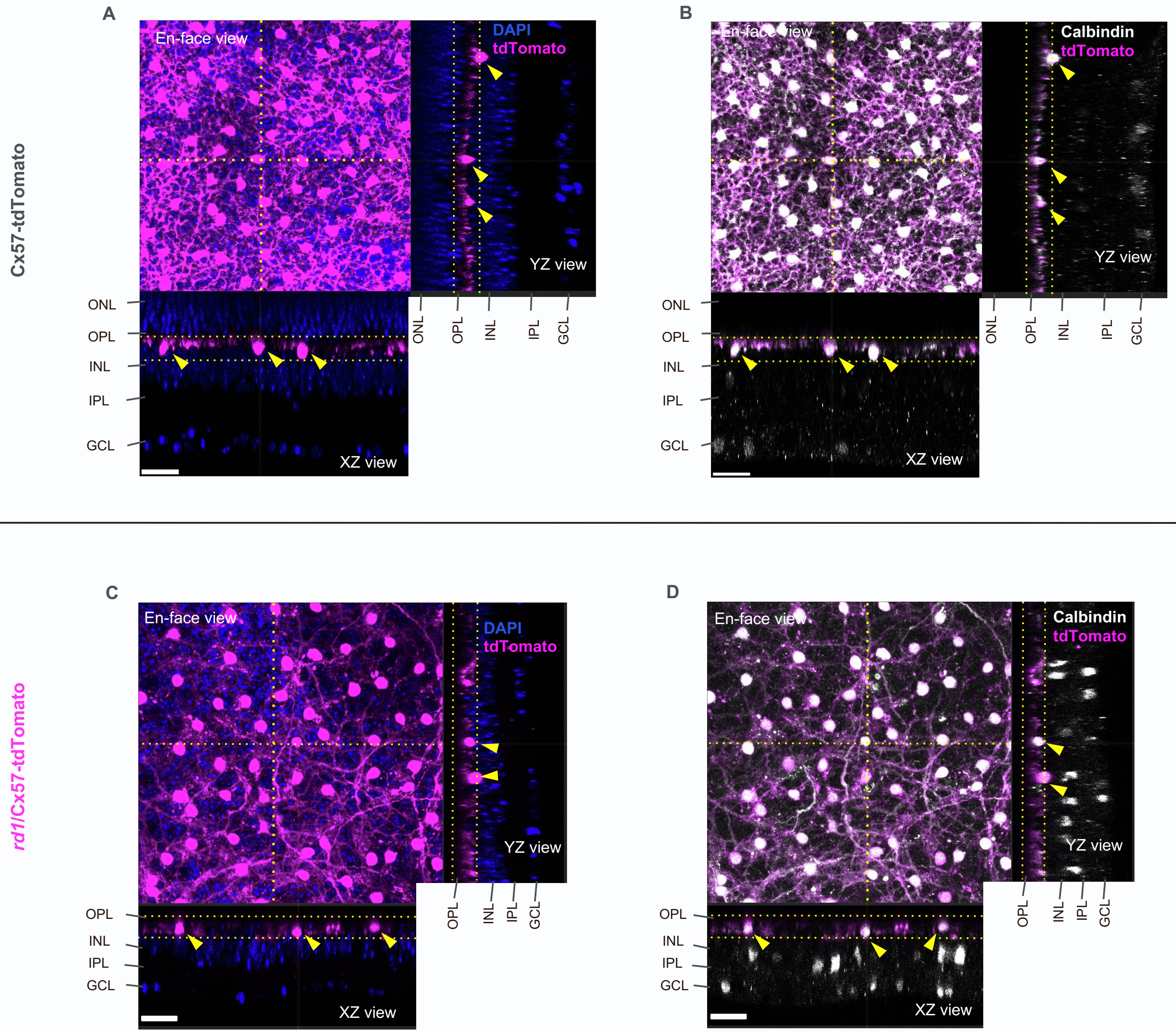

Figure S1. Fluorescent labeling was specifically applied to horizontal cells using Ai9
(A–D) Horizontal cells in WT (Cx57-tdTomato, 52 weeks; A, B) and *rd1* (*rd1*/Cx57-tdTomato, 53 weeks; C, D) mouse retinas were fluorescently labeled with tdTomato (tdTomato expression in the Ai9 reporter line), and colocalization with calbindin-positive cells along the outer plexiform layer (OPL) confirmed their identity. Yellow arrowheads indicate the horizontal cells. Calbindin-positive but tdTomato-negative cells were observed in the inner nuclear layer (INL), which may represent amacrine cells. The yellow dotted lines in each dimensional view (XY, XZ, YZ) indicate the corresponding positions where the maximum intensity projections were generated along the perpendicular cross-sectional planes. Scale bars: 30 μm.
ONL; outer nuclear layer, OPL; outer plexiform layer, INL; inner nuclear layer, IPL; inner plexiform layer, GCL; ganglion cell layer

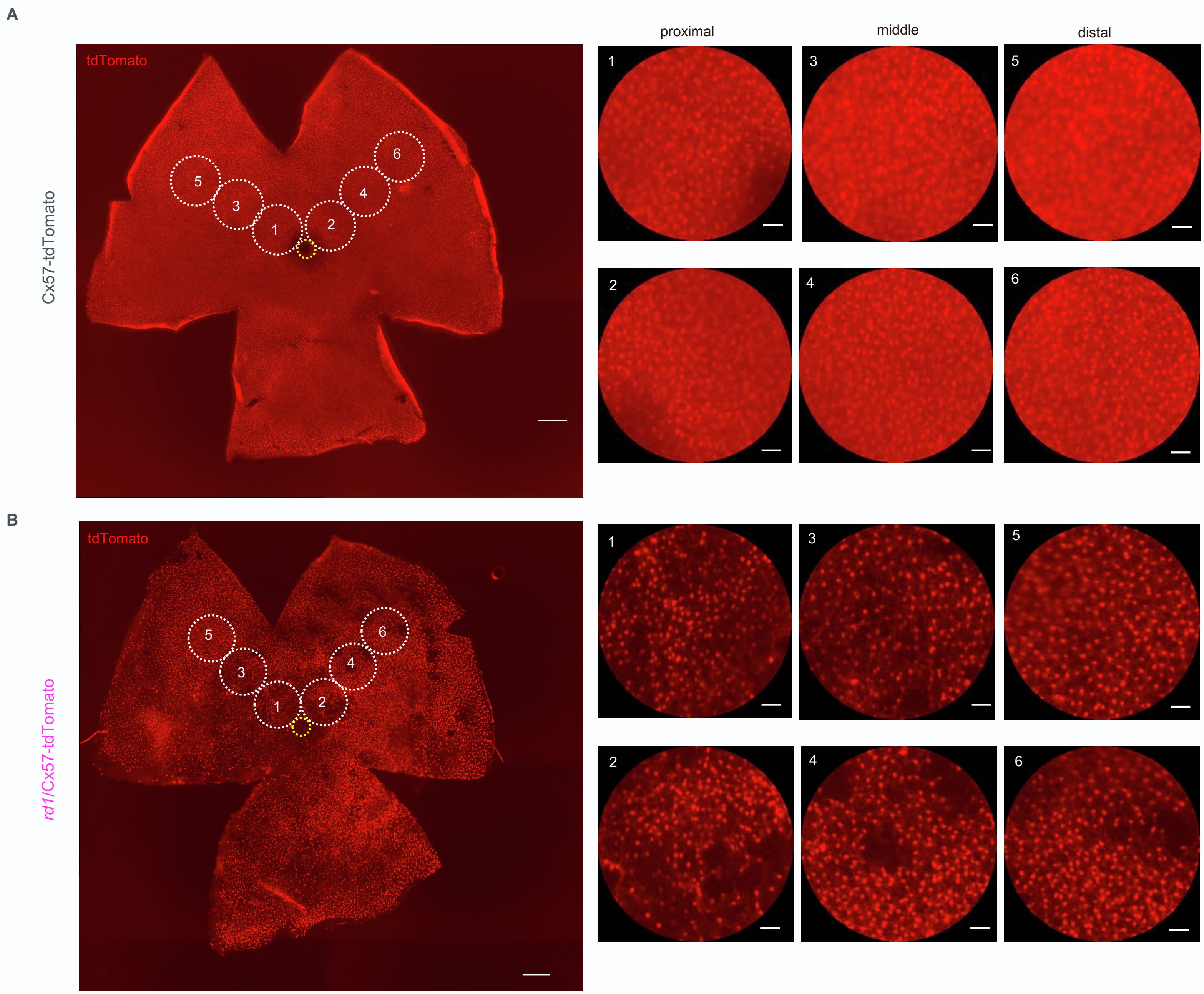

Figure S2. Representative images of 20-week-old horizontal cell degeneration in Cx57-tdTomato and *rd1*/Cx57-tdTomato retinas, related to Figure 1.
(A and B) Representative images of horizontal cells in 20-week-old Cx57-tdTomato (A) and *rd1*/Cx57-tdTomato (B) retinas. Horizontal cells (HCs) were labeled with Cx57-tdTomato. On the right, the regions marked by circles 1–6 are magnified and categorized as proximal, middle, or distal relative to the optic disc. The yellow circle indicates the optic disc. Scale bars (left image) 300 µm, (right magnified images) 30 µm.

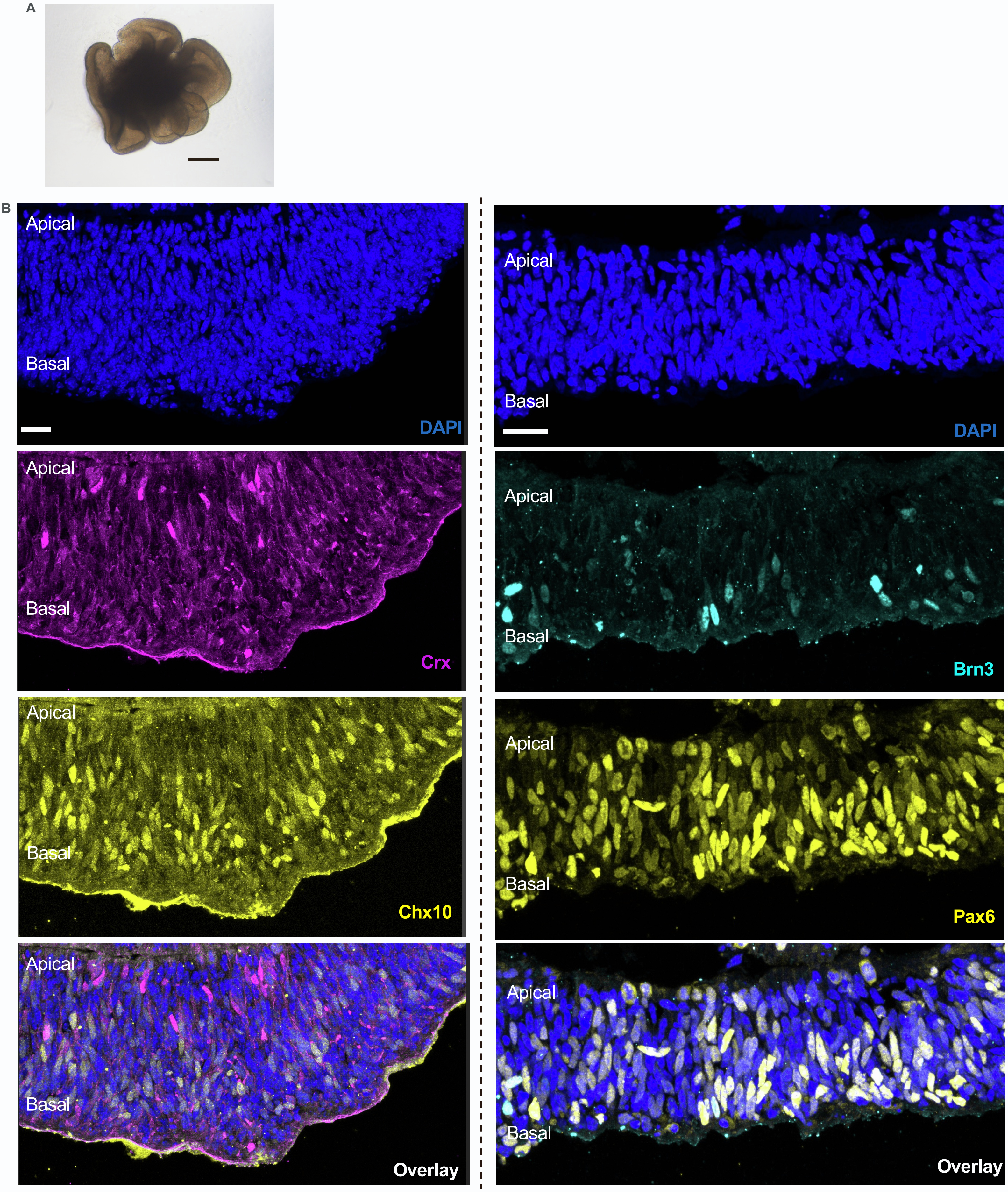

Figure S3. Immunohistochemical analysis of DD13 retinal organoids derived from Islet1 knockout mouse ES cells at the time of transplantation

(A) Representative image of retinal organoid at day 13 of differentiation (DD13). Scale bar: 200 µm.

(B) Representative immunostainings of retinal organoids at DD13 with antibodies against Crx (photoreceptor precursor marker), Chx10 (retinal progenitor marker), Brn3 (retinal ganglion cell marker), and Pax6 (retinal progenitor marker). Scale bars: 20 µm.

A

TP-*rd1*

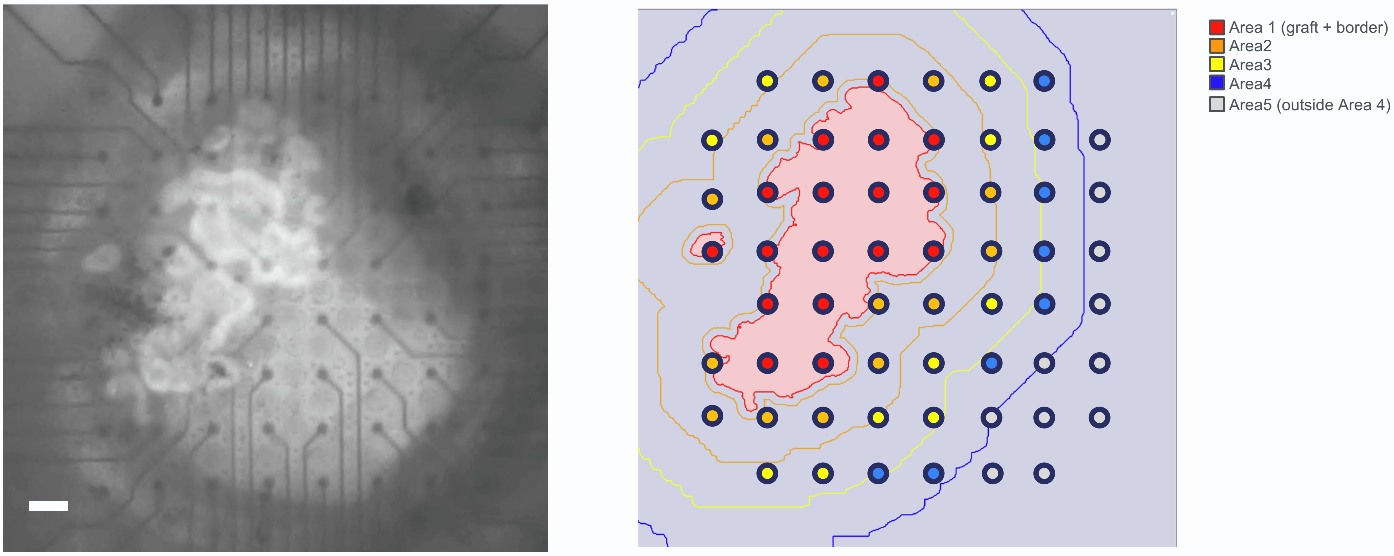

rd1

B

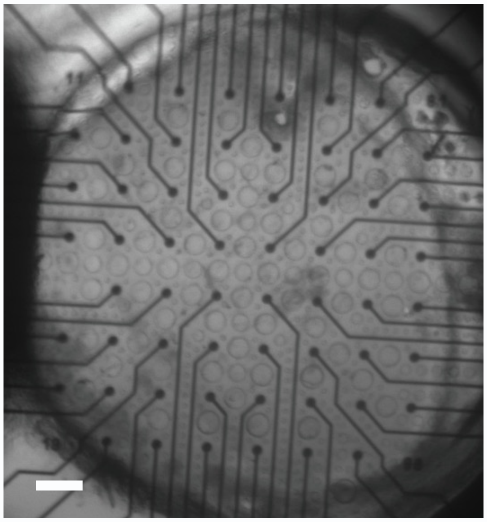

Figure S4. Representative images of TP-*rd1* and *rd1* retinas during MEA recording related to Figure 4.
(A) Left view: The TP-*rd1* retina was placed with the RGC side attached to the MEA, and the grafted area was identified by the presence of Nrl-CtBP2:tdTomato fluorescence. Right view: MEA areas were divided into Area 1 (graft and border) and Areas 2–5 as described in Experimental procedures. Scale bar: 200 μm.
(B) *rd1* retina was placed with the RGC side attached to the MEA. Scale bar: 200 μm.

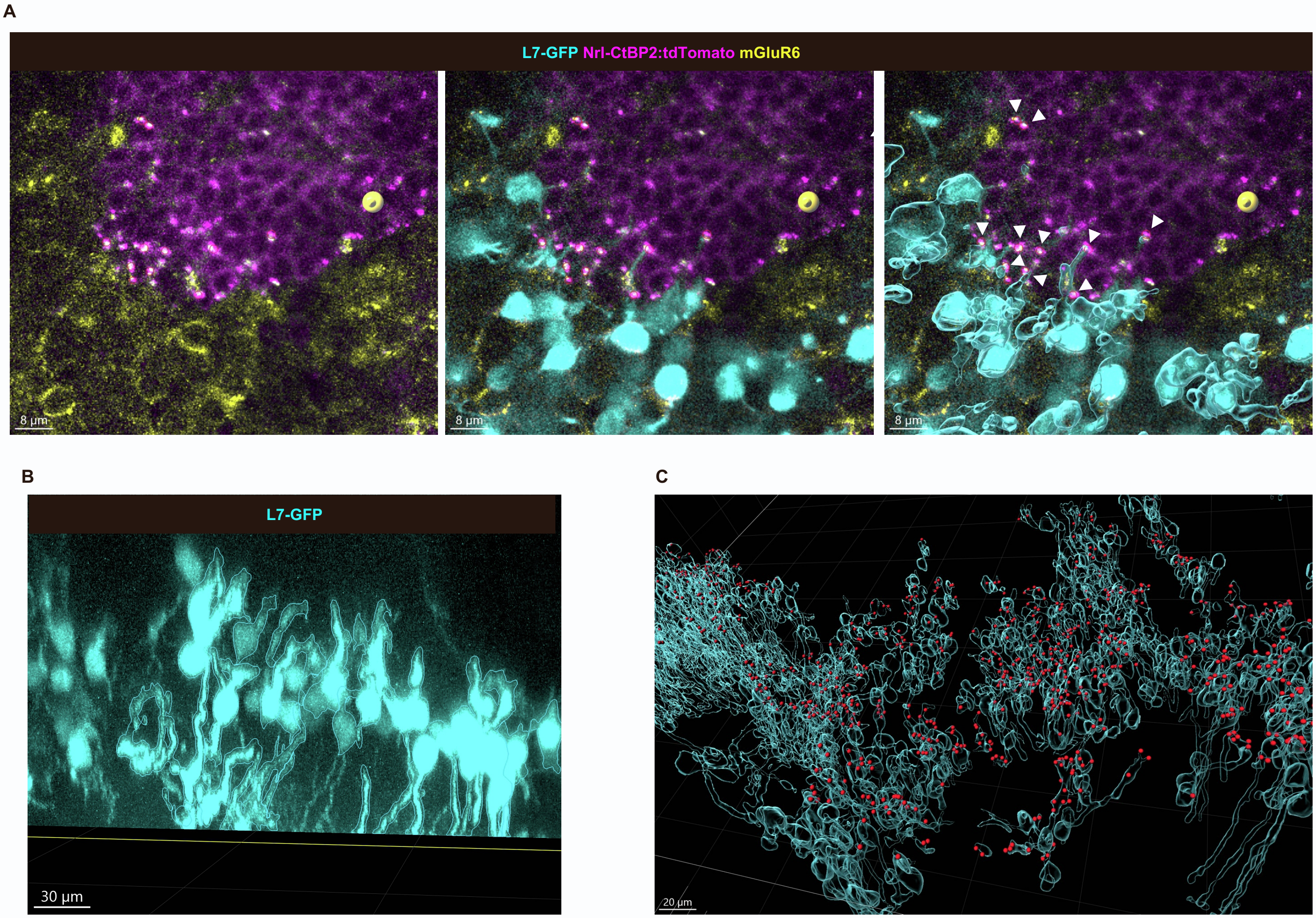

Figure S5. Example of synapse detection, related to Figure 5

(A) Host-graft synapses displayed on a representative sectional view of a 3-dimensional (3D) reconstructed immunohistochemical image. Using Imaris software on 3D images, host-graft synapses were identified as CtBP2 spots (magenta) located within 1.5 µm of both mGluR6-positive spots (yellow) and L7-GFP-positive host rod bipolar cell (RBC) dendrites (cyan). Identified synapses are indicated by white arrowheads.

(B) Tracing of L7-GFP-positive RBCs (blue contours).

(C) Host-graft synapses (red spots) detected in close contact with host RBC dendrites in 3D view.

Table S1. Summary of non-transplanted retinas used in the experiments

Mouse-ID	Gene	Age at analysis (Weeks)	Related figure	Related analysis
rd1-1	*rd1*/L7-GFP/Cx57-tdTomato	20	Figure1	HC count *
rd1-2	*rd1*/L7-GFP/Cx57-tdTomato	20	Figure1	HC count
rd1-3	*rd1*/L7-GFP/Cx57-tdTomato	20	Figure1	HC count
rd1-4	*rd1*/L7-GFP/Cx57-tdTomato	53	Figure1	HC count
rd1-5	*rd1*/L7-GFP/Cx57-tdTomato	53	Figure1	HC count
rd1-6	*rd1*/L7-GFP/Cx57-tdTomato	53	Figure1	HC count
rd1-7	*rd1*/L7-GFP	10	Figure3	IHC
rd1-8	*rd1*	6	Figure4	MEA
rd1-9	*rd1*	4	Figure4	MEA
rd1-10	*rd1*	4	Figure4	MEA
Cx57-tdTomato-1	Cx57-tdTomato	22	Figure1	HC count
Cx57-tdTomato-2	Cx57-tdTomato	20	Figure1	HC count
Cx57-tdTomato-3	Cx57-tdTomato	21	Figure1	HC count
Cx57-tdTomato-4	Cx57-tdTomato	53	Figure1	HC count
Cx57-tdTomato-5	Cx57-tdTomato	53	Figure1	HC count
Cx57-tdTomato-6	Cx57-tdTomato	53	Figure1	HC count
rd1-dHC	*rd1*-dHC	10	Figure3	IHC

*HC count refers to the quantification of horizontal cells.

Table S2. Summary of each transplanted retina used for the experiment

Mouse-ID	Host	Age at time of transplantation (Weeks)	Post-TP (weeks)	Age at analysis (Weeks)	Related figure	Related analysis
TP-*rd1*-1	*rd1*/L7-GFP/Cx57-tdTomato	20.0	8.7	28.7	Figure2	IHC
TP-*rd1*-2	*rd1*/L7-GFP/Cx57-tdTomato	14.3	7.7	22.0	Figure2, FIgure4	IHC, MEA
TP-*rd1*-3	*rd1*/L7-GFP	10.1	8.3	18.4	Figure4	MEA
TP-*rd1*-4	*rd1*/L7-GFP	10.1	9.0	19.1	Figure4	MEA
TP-*rd1*-5	*rd1*/L7-GFP	10.1	8.7	18.9	Figure4	MEA
TP-*rd1*-6	rd1/L7-GFP/Cx57-tdTomato	14.3	8.1	22.4	Figure4	MEA
TP-*rd1*-7	*rd1*/L7-GFP	10.1	8.9	19.0	Figure4	MEA
TP-*rd1*-8	*rd1*/L7-GFP	10.1	9.7	19.9	Figure4	MEA
TP-*rd1*-9	*rd1*/L7-GFP	10.7	8.0	18.7	Figure4, Figure5	MEA, Synapse quantification
TP-*rd1*-10	*rd1*/L7-GFP	18.9	6.0	24.9	FIgure4, Figure5	MEA, Synapse quantification
TP-*rd1*-11	*rd1*/L7-GFP	13.7	19.3	33.0	Figure5	Synapse quantification
TP-*rd1*-12	*rd1*/L7-GFP	29.9	9.7	39.6	Figure5	Synapse quantification
TP-*rd1*-dHC-1	*rd1*-dHC	14.3	8.0	22.3	Figure4	MEA
TP-*rd1*-dHC-2	*rd1*-dHC	14.3	8.3	22.6	Figure4	MEA
TP-*rd1*-dHC-3	*rd1*-dHC	14.3	8.7	23.0	Figure4	MEA
TP-*rd1*-dHC-4	*rd1*-dHC	20.0	8.3	28.3	Figure4	MEA
TP-*rd1*-dHC-5	*rd1*-dHC	20.0	9.0	29.0	Figure4	MEA
TP-*rd1*-dHC-6	*rd1*-dHC	11.7	7.9	19.6	Figure4	MEA
TP-*rd1*-dHC-7	*rd1*-dHC	11.3	7.7	19.0	Figure5	MEA, Synapse quantification
TP-*rd1*-dHC-8	*rd1*-dHC	16.1	11.0	27.1	Figure5	Synapse quantification
TP-*rd1*-dHC-9	*rd1*-dHC	13.9	9.0	22.9	Figure5	Synapse quantification
TP-*rd1*-dHC-10	*rd1*-dHC	13.9	9.0	22.9	Figure5	Synapse quantification
TP-*rd1*-dHC-11	*rd1*-dHC	11.7	7.9	19.6	Figure6	IHC
TP-*rd1*-dHC-12	*rd1*-dHC	18.3	5.3	23.6	Figure6	Electron microscopy

Table S3. Summary of light-evoked RGCs, related to Figure 4

Gene	Mapping area	Low signal	Not classified	OFF	ON	ON-OFF	Total number of RGCs
TP-*rd1*	1	50	91	3	38	25	207
	2	22	82	4	20	7	135
	3	14	96	0	8	3	121
	4	14	59	0	5	0	78
	5	1	23	0	0	0	24
TP-*rd1*-dHC	1	27	79	2	12	5	125
	2	19	105	2	8	1	135
	3	16	87	1	2	0	106
	4	10	75	0	4	0	89
	5	8	56	0	0	0	64

Supplemental procedures

Immunostaining of DD13 retinal organoids

For immunostaining of DD13 retinal organoids, organoids were fixed in 4 % paraformaldehyde (Wako Pure Chemical Industries, 30525-89-4) for 15 min at RT, cryoprotected in 30 % sucrose, embedded in optimal cutting temperature (OCT) compound, and sectioned at a thickness of 10 μm using a cryostat. Sections were washed with PBS and incubated in a blocking buffer (3 % Triton X-100 and 1 % bovine serum albumin [Sigma, A4503]) for 1 h at RT. The sections were then incubated with primary antibodies diluted 1:1000 for 24 h at 4 °C: mouse anti-Chx10 (Santa Cruz, sc365519), rabbit anti-Crx (Takara, M231), goat anti-Brn3 (Santa Cruz, sc-6026), and mouse anti-Pax6 (BD, #561462). After three washes in blocking buffer, sections were incubated with the following secondary antibodies (1:1000) for 24 h at 4 °C: donkey-anti-goat Alexa Fluor 488 (Invitrogen, A11055), donkey-anti-mouse Alexa Fluor 647 (Invitrogen, A31571), and donkey-anti-rabbit Alexa Fluor 546 (Invitrogen, A10040), and DAPI (Invitrogen, 1:1000, D1306). After secondary antibody incubation, the sections were washed thrice with PBS and mounted using Vectashield (Vector Laboratories, H-1000). Images were acquired using a Leica TCS SP8 confocal microscope and processed using the Imaris Microscopy Image Analysis Software (Oxford Instruments, http://www.bitplane.com/).

Spike sorting

The raw data were band-pass filtered to extract components between 100 and 3,000 Hz. A maximum negative value exceeding the threshold, defined by the following formula, was detected as the spike (Quiroga et al., 2004).

$$\text{threshold} = -4 \times \text{median}(|x|0.6745)$$

where x denotes a data point. The first and second differences between the data points were

calculated from the data points between 1 and 2 ms from the peak of the detected spike, and the dimensions were converted into two dimensions using principal component analysis. Clustering was performed using density-based spatial clustering of applications with noise as a feature (HDBSCAN) for each waveform. Furthermore, using a template-matching method (Zhang et al., 2004), we implemented an algorithm to merge clusters and rescue spikes from a group of waveforms considered as noise into one of the clusters. To verify the accuracy of spike sorting, we performed autocorrelation analysis on the sorted spike train from each unit and confirmed the presence of a refractory period (± 1 ms).

Supplemental references

Quiroga, R.Q., Nadasdy, Z., and Ben-Shaul, Y. (2004). Unsupervised spike detection and sorting with wavelets and superparamagnetic clustering. Neural Comput. 16, 1661–1687. 10.1162/089976604774201631.

Zhang, P.M., Wu, J.Y., Zhou, Y., Liang, P.J., and Yuan, J.Q. (2004). Spike sorting based on automatic template reconstruction with a partial solution to the overlapping problem. J. Neurosci. Methods 135, 55–65. 10.1016/j.jneumeth.2003.12.001.
